# Identification of novel biomarkers linking depressive disorder and Alzheimer’s disease based on an integrative bioinformatics analysis

**DOI:** 10.1186/s12863-023-01120-x

**Published:** 2023-04-15

**Authors:** Jin Song, Zilong Ma, Huishi Zhang, Ting Liang, Jun Zhang

**Affiliations:** 1 Out-Patient Department, Wuhan Mental Health Center, Wuhan, 430012 Hubei Province China; 2 Out-Patient Department, Wuhan Hospital for Psychotherapy, Wuhan, 430012 Hubei Province China; 3 Ward of Sleep Disorders, Wuhan Mental Health Center, Wuhan, 430012 Hubei Province China; 4 Ward of Sleep Disorders, Wuhan Hospital for Psychotherapy, Wuhan, 430012 Hubei Province China; 5 Research Center for Psychological and Health Sciences, China University of Geosciences, Wuhan, Hubei Province 430012 China; 6 National Medical Institution Conducting Clinical Trials Office, Wuhan Mental Health Center, Wuhan, 430012 Hubei Province China; 7 National Medical Institution Conducting Clinical Trials Office, Wuhan Hospital for Psychotherapy, Wuhan, 430012 Hubei Province China; 8 Ward of Traditional Chinese Medicine, Wuhan Mental Health Center, Wuhan, 430012 Hubei Province China; 9 Ward of Traditional Chinese Medicine, Wuhan Hospital for Psychotherapy, Wuhan, 430012 Hubei Province China

**Keywords:** Depressive, Bioinformatics, Immune cells, Alzheimer’s disease, Immune-related genes

## Abstract

**Background:**

Previous reports revealed that a history of major depressive disorder (MDD) increased the risk of Alzheimer’s disease (AD). The immune disorder is associated with MDD and AD pathophysiology. We aimed to identify differentially expressed immune-related genes (DEIRGs) that are involved in the pathogenesis of MDD and AD.

**Methods:**

We downloaded mRNA expression profiles (GSE76826 and GSE5281) from the Gene Expression Omnibus (GEO) database. The R software was used to identify DEIRGs for the two diseases separately. Functional enrichment analysis and PPI network of DEIRGs were performed. Finally, the relationship between shared DEIRGs and immune infiltrates of AD and MDD were analyzed, respectively.

**Results:**

A total of 121 DEIRGs linking AD and MDD were identified. These genes were significantly enriched in immune-related pathways, such as the JAK-STAT signaling pathway, regulation of chemotaxis, chemotaxis, cytokine-cytokine receptor interaction, and primary immunodeficiency. Furthermore, three shared DEIRGs (IL1R1, CHGB, and NRG1) were identified. Correlation analysis between DEIRGs and immune cells revealed that IL1R1 and NRG1 had a negative or positive correlation with some immune cells both in AD and MDD.

**Conclusion:**

Both DEIRGs and immune cell infiltrations play a vital role in the pathogenesis of AD and MDD. Our findings indicated that there are common genes and biological processes between MDD and AD, which provides a theoretical basis for the study of the comorbidity of MDD and AD.

## Introduction

Major depressive disorder (MDD) is a common neuropsychiatric disorder, with high suicide mortality and morbidity [1]. The loss of interest and persistent depression are the major clinical manifestations of MDD [2]. This disease impacts approximately 163 million people worldwide [3]. Unfortunately, the potential pathophysiological mechanisms are still unclear. The etiology of MDD is complex, and the interaction of intestinal microbes, immunity, neuroendocrine, genetics, psychosocial environment and other factors are associated with the pathogenesis of MDD [4, 5]. Emerging evidence showed that immune disorder, specifically inflammatory responses, is related to symptoms of MMD [6]. It has been reported that depression involves changes in multiple aspects of the immune system that may promote the progression of various psychiatric disorders, including MDD [7]. Another neurodegenerative disorder, Alzheimer’s disease (AD), is the most common form of neurodegenerative dementia in old age [8]. Although the exact pathogenesis of AD is not well understood, researchers have reported that the interplay of genetic and environmental factors may be involved in the initiation of AD pathogenesis [9, 10]. The immune system is now considered an important role in AD. Recent studies have revealed the active role of brain innate immunity in AD pathogenesis progression [11, 12]. Furthermore, the genome-wide association studies and pathway analyses revealed the important role of the neuroinflammation and innate immune system in AD pathogenesis [13–15]. A recent study highlighted that the ability to modulate target neuroinflammation and neuroimmune interactions is a promising opportunity in the search for optimal therapies for AD [16]. The peripheral immune changes are associated with cognitive dysfunction, and Tfh and IL-21 may be developed as novel circulatory biomarkers for AD [17].

Recent reports have revealed that there is a strong relationship between MDD and AD [18, 19]. For example, a systematic analysis result revealed shared pathways and biological processes between MDD and AD and offered a hint for the comorbidity of MDD and AD [20]. Depression could act as a risk factor for the late development of AD [21]. The long-term depressive symptoms contribute to mild cognitive impairment turns into AD [22, 23]. Besides, neurodegenerative phenomena were also found in the hippocampus of MDD patients [24]. Furthermore, recent reports have revealed that the cascades and molecular mechanisms of MDD, including chronic neuroinflammation, impairment of neurotrophin signaling, and immune dysregulation, are also implicated in the pathogenesis of AD [25–28]. These findings implied that immune dysregulation may be the common pathogenesis of MDD and AD. Therefore, the study of immune responses, inflammatory processes, and their association with each other, is essential for a deeper understanding of AD and MDD pathogenesis [29]. However, few researchers have reported the immune-related genes (IRGs) that are implicated in both disorders.

In the present study, we identified the IRGs related to MDD and AD via bioinformatics methods. And we performed the functional enrichment analysis via Metascape. Moreover, the relationship between shared IRGs and immune cell infiltrates was assessed to better understand the common immune mechanism of MDD and AD.

## Materials and methods

### Collection of transcriptome data from GEO

The GEOquery package was used to download the GSE76826, GSE5281, GSE98793, and GSE132903 datasets from the Gene Expression Omnibus (GEO) [30]. The GSE76826 dataset contains 12 healthy individuals and 20 MDD patients. The GSE5281 dataset contains 74 healthy individuals and 87 AD patients. The GSE98793 dataset contains 64 MDD patients with generalized anxiety disorder and 64 healthy individuals. GSE132903 contains 97 AD patients and 98 healthy individuals. GSE76826 and GSE5281 were used as test cohorts. GSE98793 and GSE132903 were used as validation cohorts. The detailed sample information was presented in Table 1.

**Table 1:** GEO microarray datasets

GEO ID	Platform	Control group	Disease group	Source	Application
GSE76826	GPL170177	12 healthy individuals	20 MDD patients	Blood	Analysis
GSE5281	GPL570	74 healthy individuals	87 AD patients	Brain tissue	Analysis
GSE98793	GPL570	64 healthy individuals	64 MDD patients	Blood	Verification
GSE132903	GPL10558	98 healthy individuals	97 AD patients	Brain tissue	Verification

### Identification of differentially expressed IRGs (DEIRGs) in AD and MDD

First, the “limma” package of R software was used to identify differentially expressed genes in GSE76826 and GSE5281 datasets based on *p* < 0.05 and ∣logFC∣ ≥ 0.5. Then, a total of 1793 IRGs were collected from the ImmPort database (https://www.immport.org/shared/home). The DEGs intersected with the IRGs, and then the DEIRGs were obtained. The heatmaps of DEIRGs in MDD and AD were drawn via the ComplexHeatmap package of R software.

### Functional enrichment and protein–protein interaction (PPI) network analysis

We applied the Metascape platform (http://metascape.org) to the potential signaling pathways and biological processes of DEIRGs significantly associated with AD and MD [31]. The DEIRGs were input into the Metascape platform for KEGG and GO enrichment analysis and the parameter selected was “*Homo sapiens*” and “*p* < 0.05” [32].

#### Receiver operating characteristic analysis (ROC)

The “pROC” package of R was applied to perform the ROC curve analysis to assess the diagnostic value of shared DEIRGs [33].

#### Gene set enrichment analysis (GSEA)

GSEA was carried out to identify differences in the enrichment of pathways in shared DEIRGs between normal and disease groups. The “clusterProfiler”, “enrichplot”, “pathwork”, and “DOSE” packages of R were used to perform GSEA. The gene set of “h.all.v7.3.symbols” was downloaded from the MsigDB database and used as a reference gene set. *P* < 0.05 was considered statistically significant.

#### Assessment of immune cell infiltration

The microenvironment Cell Populations-counter (MCPCounter) is a bioinformatics tool that assesses the proportion of different immune cells based on specific molecular markers [34]. We used the MCPCounter package of R to assess the immune cell infiltration of each group from GSE76826 and GSE5281 datasets, respectively. Then, the population abundance of 10 types of immune cells in the healthy and diseased groups was visualized in violin plots. Spearman correlation analysis was further performed on shared DEIRGs and 10 types of immune cells, and the result of the correlation between immune cells and genes were visualized in the lollipop diagram.

## Results

### Identification of DEIRGs

**Fig. 1:**
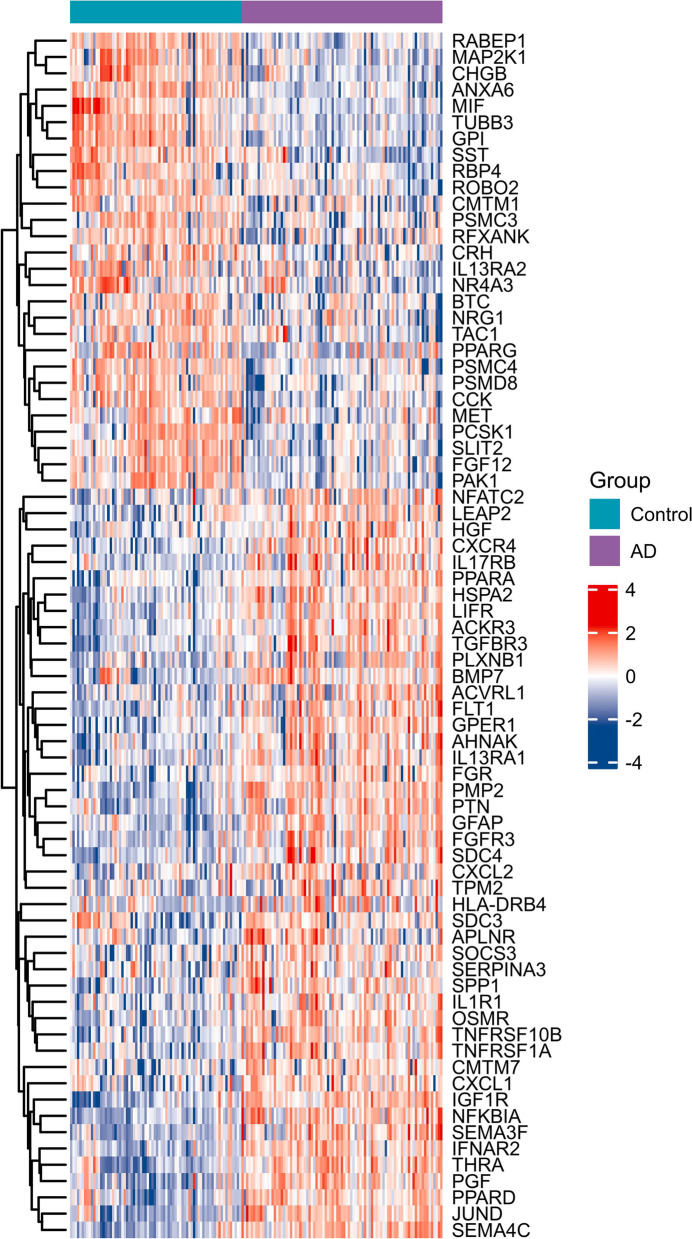
Identification of DEIRGs in the GSE5281 dataset. Heatmap of DEIRGs in AD and control samples

**Fig. 2:**
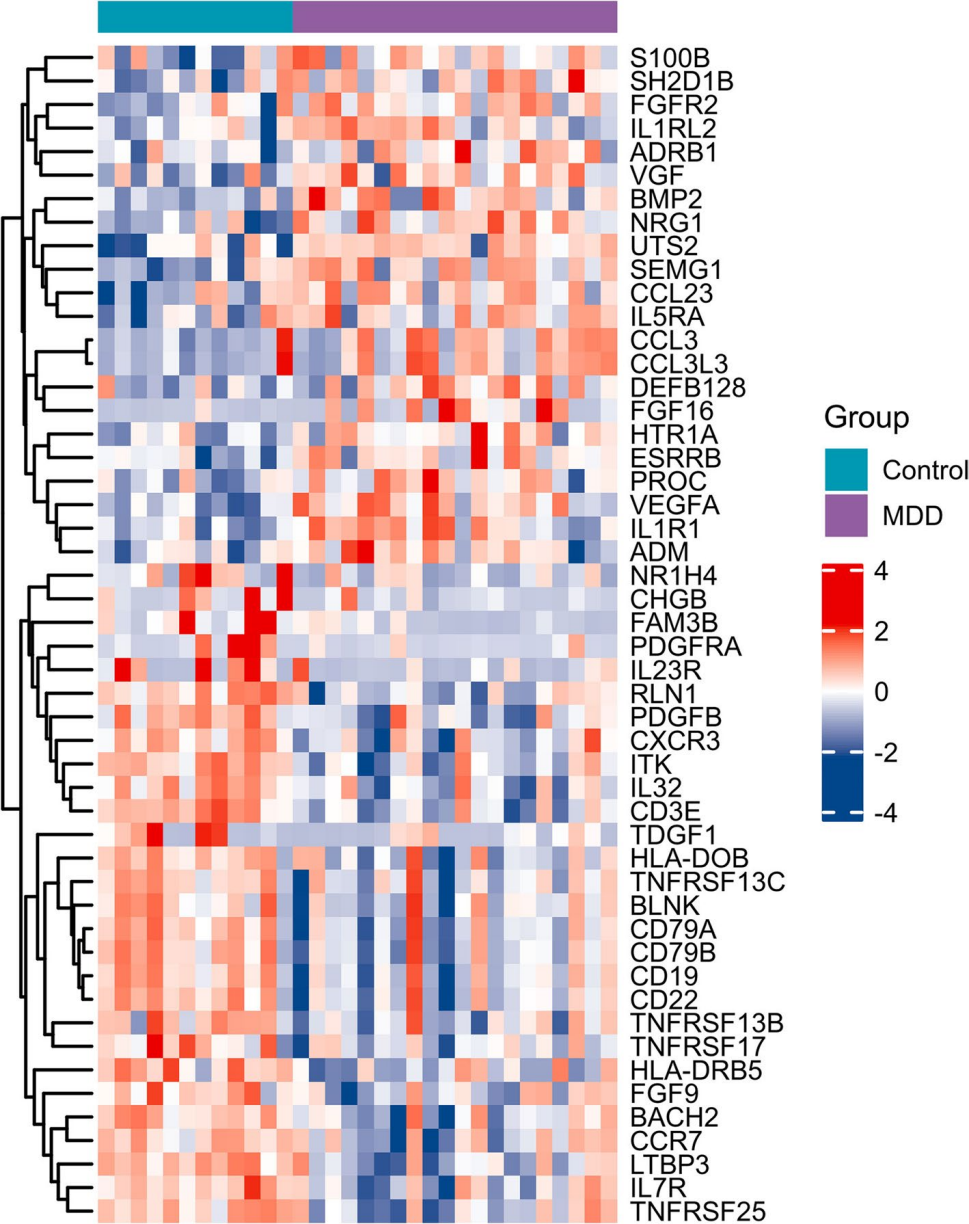
Identification of DEIRGs in the GSE76826 dataset. Heatmap of DEIRGs in MDD and control samples

After data preprocessing, a total of 74 DEIRGs were identified from the GSE5281 dataset, of which 28 genes were downregulated and 46 genes were upregulated in the AD group (Fig. 1). A total of 50 DEIRGs were identified from the GSE76826 dataset, of which 28 genes were downregulated and 22 genes were upregulated in the MDD group (Fig. 2).

### Functional enrichment analysis of DEIRGs

**Fig. 3:**
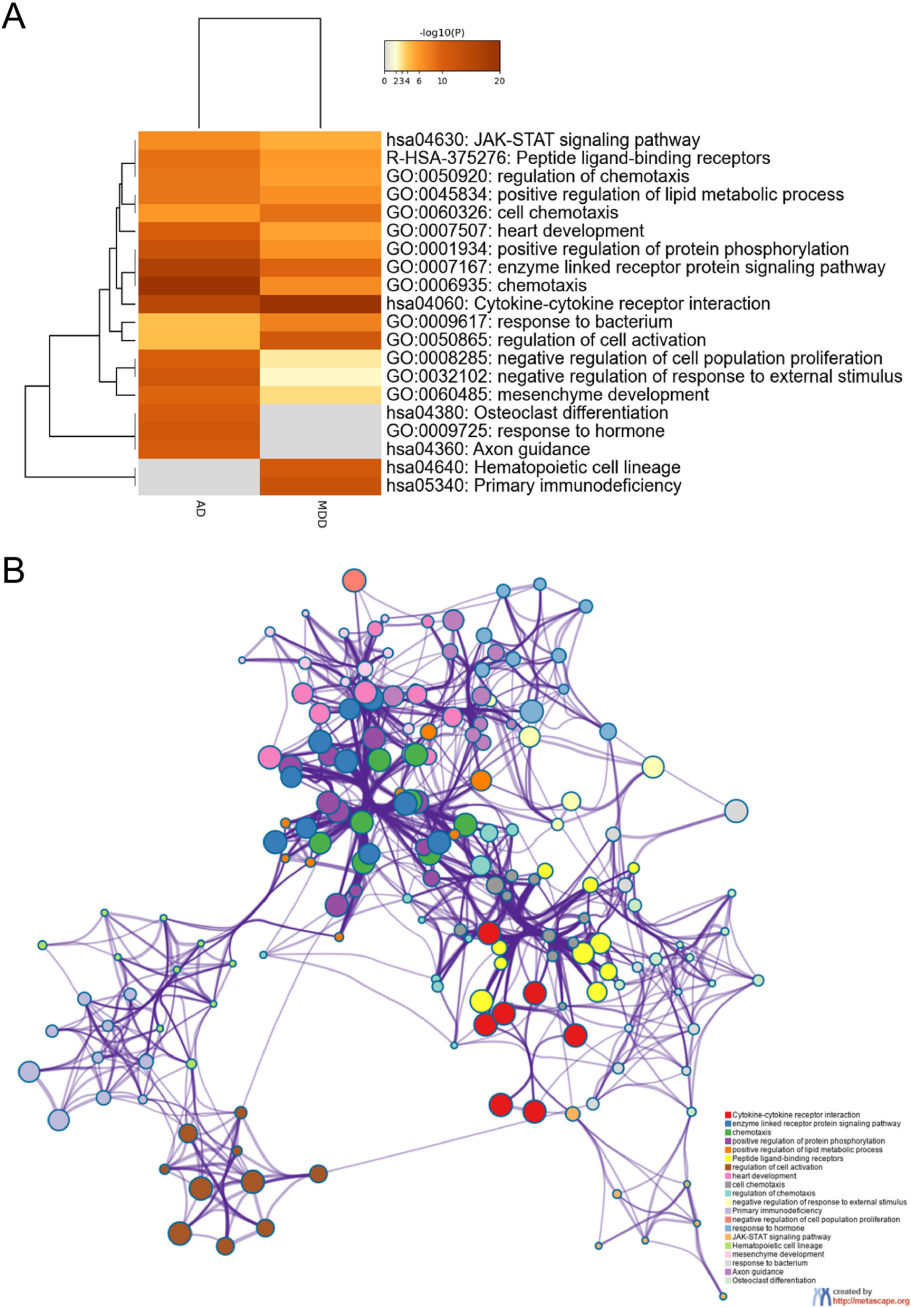
Functional enrichment analysis of DEIRGs using Metascape. **A** Heatmap of the top 20 enriched terms across targets associated with AD and MDD, colored based on the p-value. **B** Network of the top 20 enriched terms colored based on cluster ID

As shown in Fig. 3, these 121 DEIRGs were mainly enriched in primary immunodeficiency, regulation of cell activation, response to a bacterium, cytokine-cytokine receptor interaction, chemotaxis, cell chemotaxis, regulation of chemotaxis, and JAK-STAT signaling pathway, etc. The findings implied that DEIRGs were significantly enriched in immune-related pathways, which have been associated with AD and MDD.

### The PPI network analysis

**Fig. 4:**
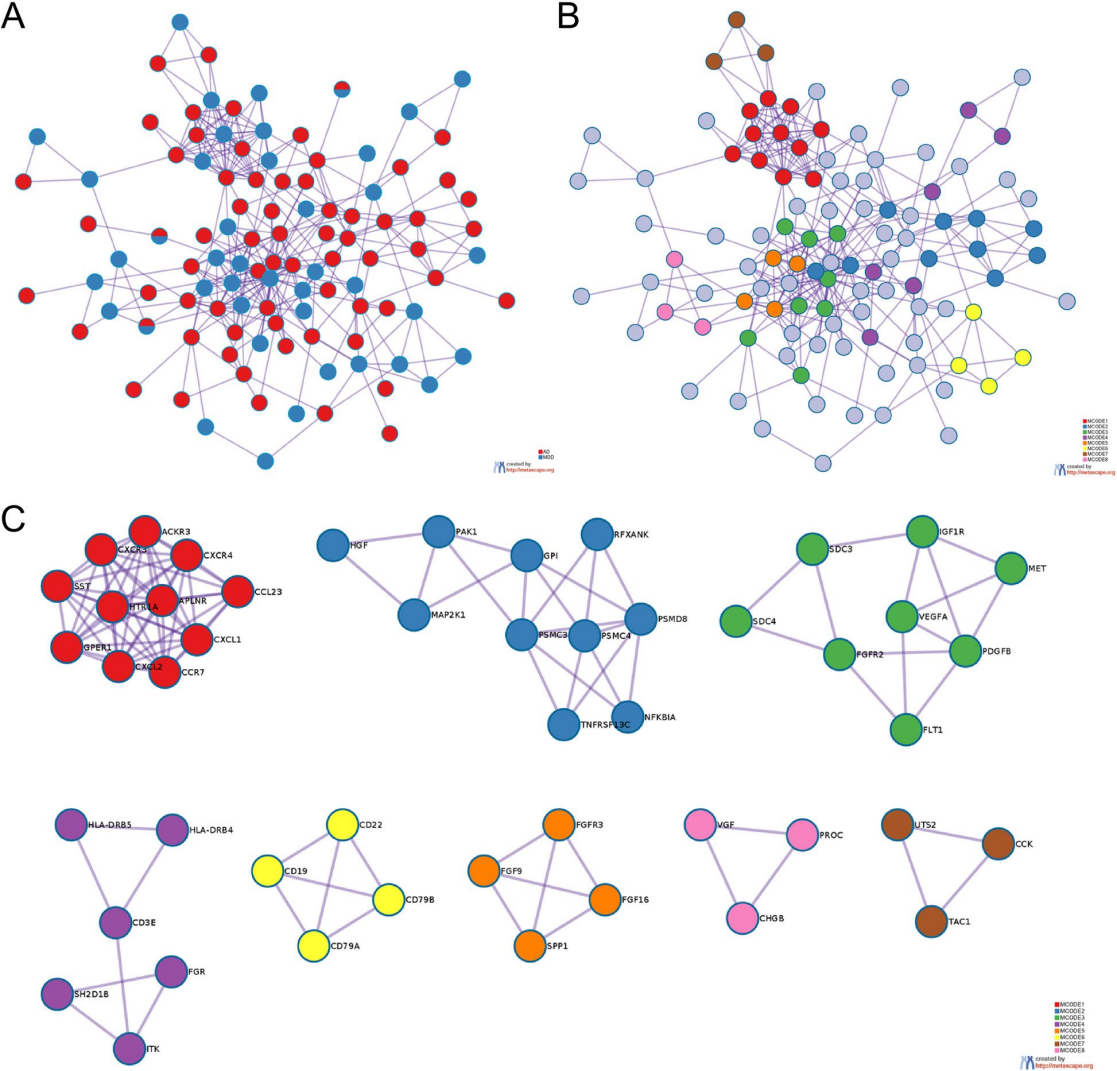
The protein–protein interaction (PPI) network analysis. **A** PPI network of the DEIRGs from AD and MDD. Red nodes were identified from the GSE5281 dataset, and blue nodes were identified from the GSE76826 dataset. **B** All lists are merged and Colored by Cluster (Full connection). **C** All lists are merged and Colored by Cluster (Keep MCODE nodes only)

Through the Metascape platform, all DEIRGs were linked to the whole protein interaction network, of which 50 genes (blue nodes) were derived from the MDD group and 74 genes (red nodes) were derived from the AD group (Fig. 4A). As shown in Fig. 4B-C, the eight different colors represent the eight module substructures identified in the molecular complex detection (MCODE) network.

### ROC curve analysis of shared DEIRGs

**Fig. 5:**
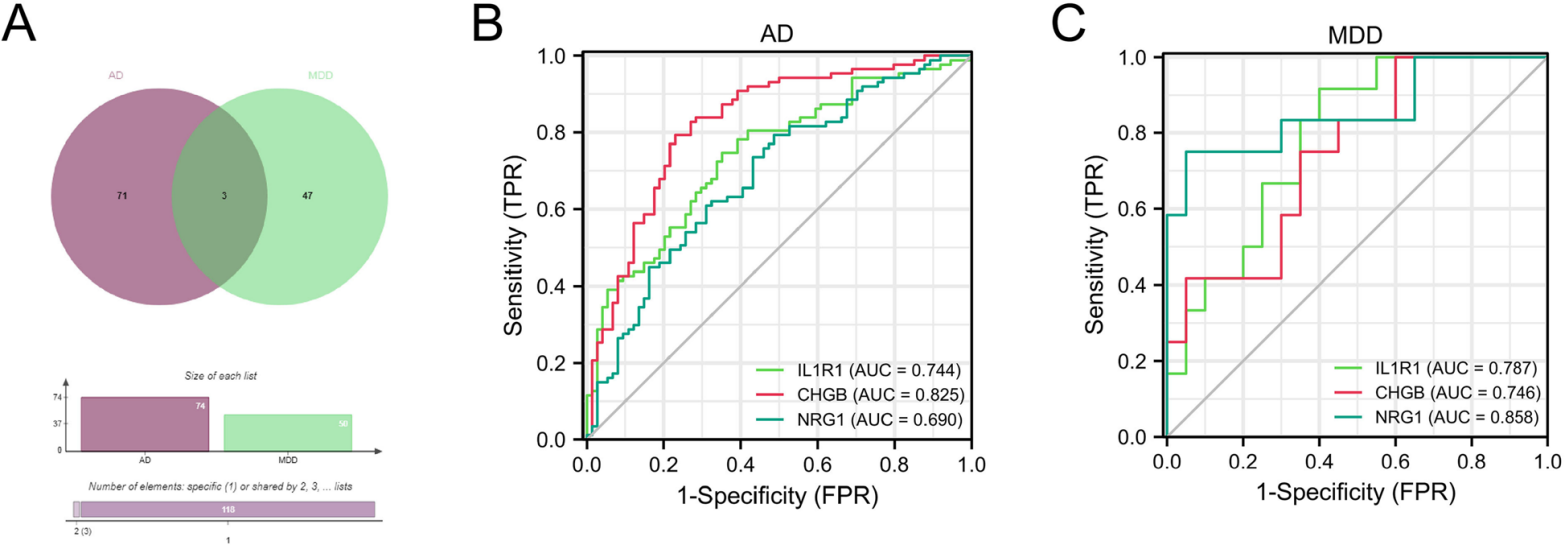
Identification of shared DEIRGs in AD and MDD. **A** Venn diagram of DEIRGs between AD and MDD. **B** ROC curve of DEIRGs in AD. **C** ROC curve of DEIRGs in MDD

**Fig. 6:**
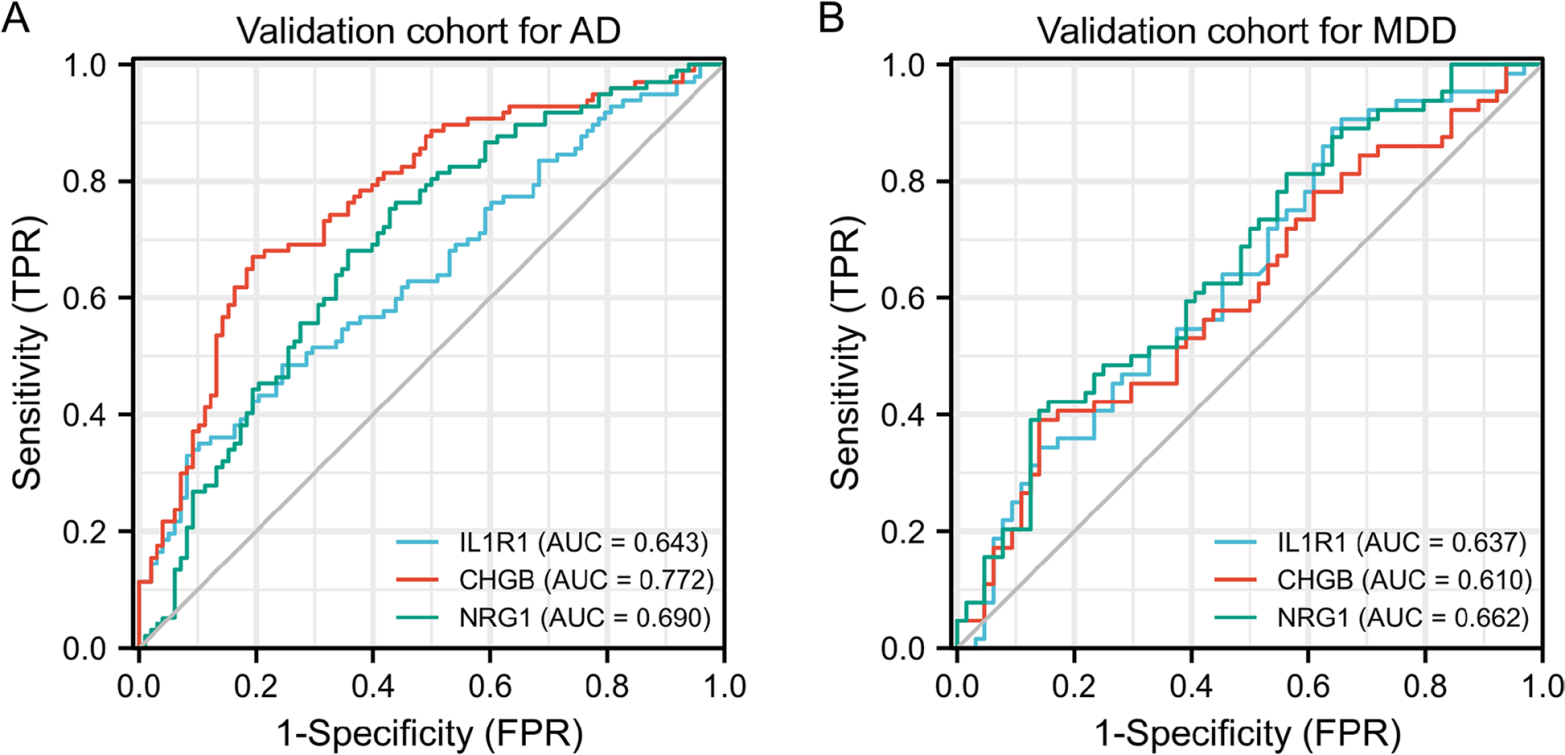
Validation of shared DEIRGs in AD and MDD. **B** ROC curve of DEIRGs in GSE132903 dataset. **C** ROC curve of DEIRGs in GSE98793 dataset

As shown in Fig. 5A, three shared DEIRGs (IL1R1, CHGB, and NRG1) were identified via the Venn tool. Furthermore, the ROC analysis indicated that IL1R1 (AUC = 0.744), CHGB (AUC = 0.825), and NRG1 (AUC = 0.69) exhibited good diagnostic values for the healthy and AD samples (Fig. 5B). As shown in Fig. 5C, IL1R1 (AUC = 0.787), CHGB (AUC = 0.746), and NRG1 (AUC = 0.858) exhibited good diagnostic values for the healthy and MDD samples. Furthermore, we used GSE98793 and GSE132903 datasets to validate these results (Fig. 6). IL1R1 (AUC = 0.643), CHGB (AUC = 0.772), and NRG1 (AUC = 0.69) had good diagnostic values for AD patients in the GSE132903 cohort (Fig. 6A). IL1R1 (AUC = 0.637), CHGB (AUC = 0.61), and NRG1 (AUC = 0.662) had good diagnostic value for MDD patients in the GSE98793 cohort (Fig. 6B). These results indicated that IL1R1, CHGB, and NRG1 had the potential diagnostic value in the diagnosis of AD and MDD patients.

### GSEA identified DEIRGs-related pathways

**Fig. 7:**
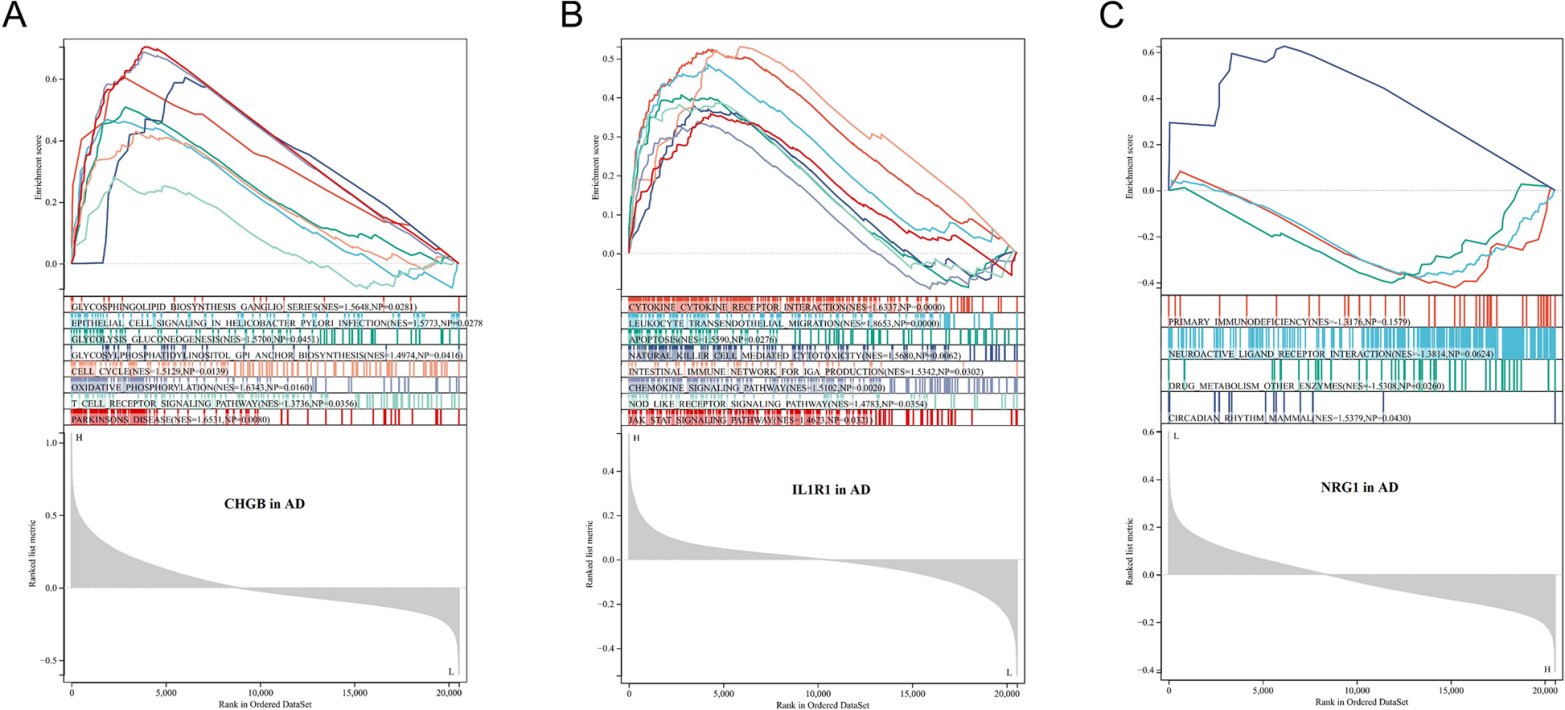
Single gene GSEA of CHGB (**A**), IL1R1 (**B**), and NRG1 (**C**) in AD

Single-gene GSEA was carried out to investigate how DEIRGs are involved in the underlying mechanisms of AD. As shown in Fig. 7A, glycosphingolipid biosynthesis ganglio series (NES = 1.56, *P* = 0.028), cell cycle (NES = 1.51, *P* = 0.013), oxidative phosphorylation (NES = 1.63, *P* = 0.016), T cell receptor signaling pathway (NES = 1.37, *P* = 0.035), and Parkinson's disease (NES = 1.65, *P* = 0.008) were significantly enriched in the CHGB high-expressed phenotype. As shown in Fig. 7B, cytokine cytokine receptor interaction (NES = 1.63, *P* < 0.001), leukocyte transendothelial migration (NES = 1.86, *P* < 0.001), apoptosis (NES = 1.55, *P* = 0.027), natural killer cell-mediated cytotoxicity (NES = 1.56, *P* = 0.006), intestinal immune network for IGA production (NES = 1.53, *P* = 0.03), chemokine signaling pathway (NES = 1.51, *P* = 0.002), NOD-like receptor signaling pathway (NES = 1.47, *P* = 0.035), and JAK-STAT signaling pathway (NES = 1.46, *P* = 0.032) were significantly enriched in the IL1R1 high-expressed phenotype. As shown in Fig. 7C, primary immunodeficiency (NES = -1.31, *P* = 0.15), neuroactive ligand-receptor interaction (NES = -1.38, *p* = 0.06), and drug metabolism other enzymes (NES = -1.53, *P* = 0.026) were enriched in the NRG1 low-expressed phenotype.

**Fig. 8:**
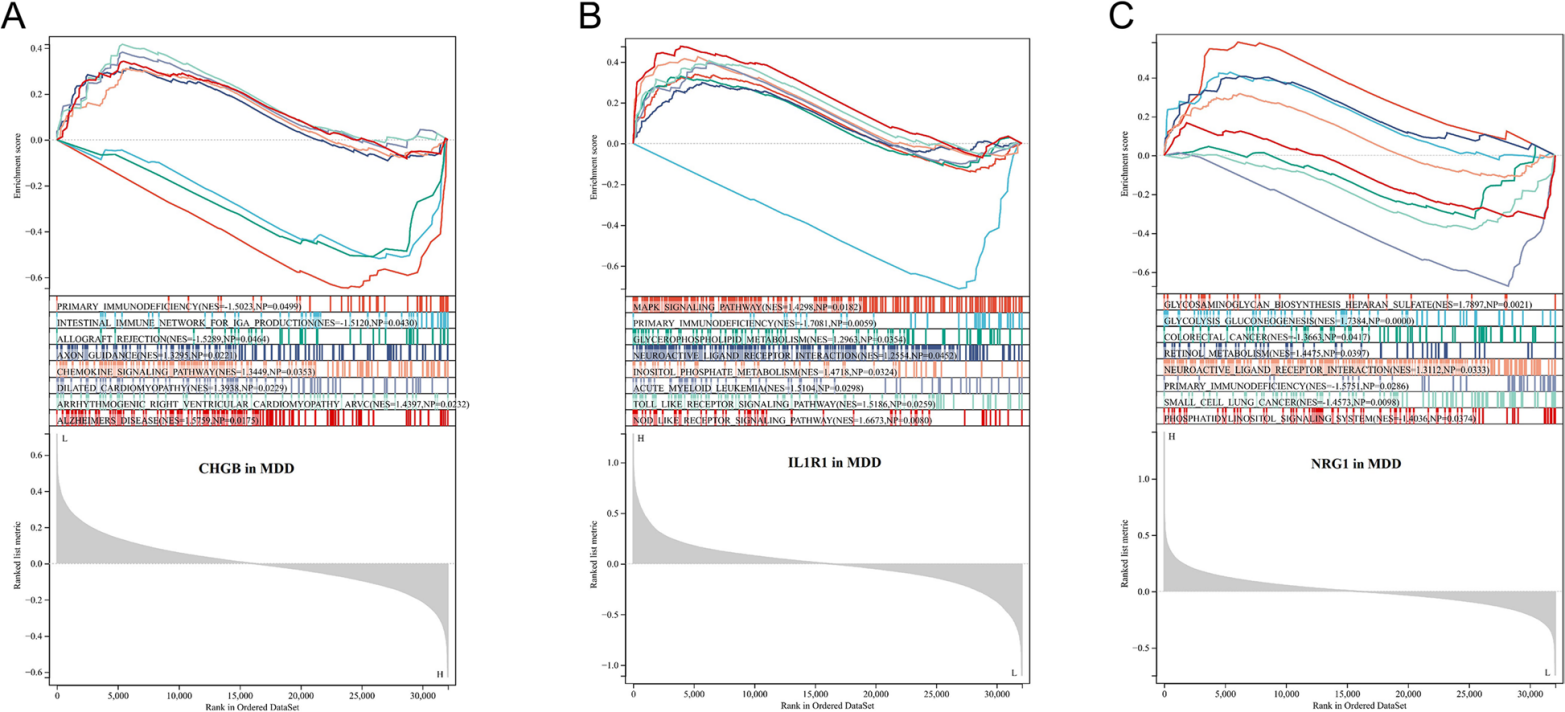
Single gene GSEA of CHGB (**A**), IL1R1 (**B**), and NRG1 (**C**) in MDD

We also performed single gene GSEA to explore the potential mechanism of MDD. As shown in Fig. 8A, primary immunodeficiency (NES = -1.5, *P* = 0.049) and intestinal immune network for IGA production (NES = -1.51, *P* = 0.043) were enriched in the CHGB high-expressed phenotype, while chemokine signaling pathway (NES = 1.34, *P* = 0.035) and Alzheimer’s disease (NES = 1.57, *P* = 0.017) were enriched in the CHGB low-expressed phenotype. As shown in Fig. 8B, MAPK signaling pathway (NES = 1.42, *P* = 0.018), neuroactive ligand-receptor interaction (NES = 1.25, *P* = 0.045), acute myeloid leukemia (NES = 1.51, *P* = 0.029), toll-like receptor signaling pathway (NES = 1.51, *P* = 0.025), and NOD-like receptor signaling pathway (NES = 1.66, *P* = 0.008) were enriched in the IL1R1 high-expressed phenotype. As shown in Fig. 8C, primary immunodeficiency (NES = -1.57, *P* = 0.028), small cell lung cancer (NES = -1.45, *P* = 0.009), and phosphatidylinositol signaling system (NES = -1.4, *P* = 0.037) were enriched in the NRG1 low-expressed phenotype. These findings suggested that the three shared genes may participate in the development of AD and MDD by impacting the immunologic processes.

### The landscape of immune infiltration in AD and MDD

**Fig. 9:**
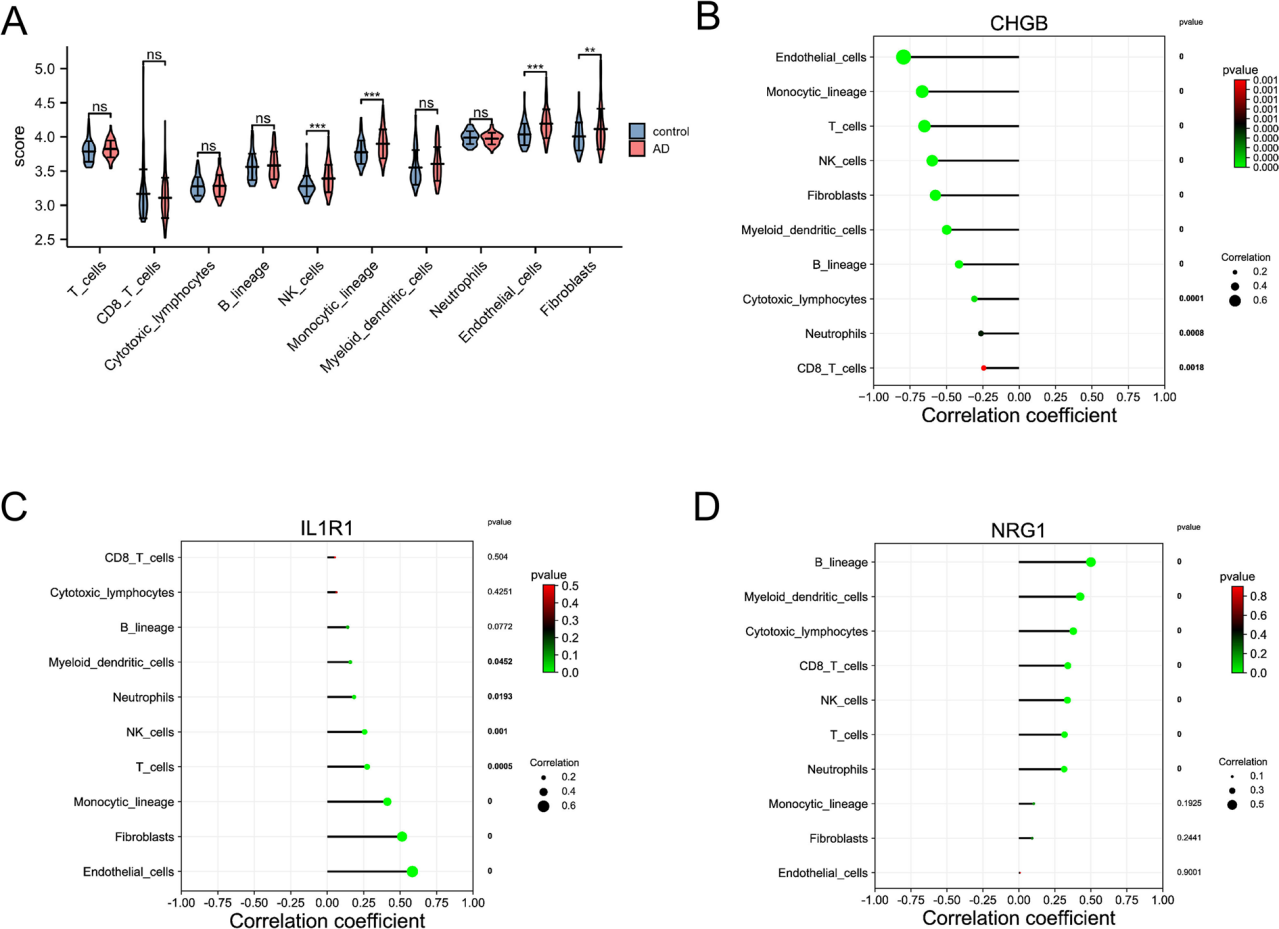
The landscape of immune cell infiltration between control and AD groups. **A** The violin plots indicate the abundance of immune cell populations in control and AD groups. Lollipop diagram of a correlation between CHGB (**B**), IL1R1 (**C**), NRG1 (**D**) expression, and immune cell infiltration level

In the present study, we used MCPCounter to assess the population abundance of two stromal (fibroblasts and endothelial cells) and eight immunes (neutrophils, myeloid dendritic cells, monocytic lineage, NK cells, B lineage, cytotoxic lymphocytes, CD8 T cells, and T cells) cells in AD, MDD and control groups. As shown in Fig. 9A, we found the differences in immune infiltrating components between AD and control groups. Our findings indicated that the abundance of NK cells, monocytic lineage, fibroblasts, and endothelial cell population in the AD group were higher than that in the control group, suggesting the immune disorder occurred in the AD group. The correlation analysis was performed to further explore the relationship between shared DEIRGs and immune cells. As shown in Fig. 9B, CHGB expression was negatively correlated with endothelial cells, monocytic lineage, T cells, NK cells, fibroblasts, myeloid dendritic cells, B lineage, cytotoxic lymphocytes, neutrophils, and CD8 T cells (all *p* < 0.01). IL1R1 was positively correlated with endothelial cells (*p* < 0.01), fibroblasts (*p* < 0.01), monocytic lineage (*p* < 0.01), T cells (*p* < 0.01), NK cells (*p* < 0.01), neutrophils (*p* < 0.05), and myeloid dendritic cells (*p* < 0.05) (Fig. 9C). NRG1 was positively correlated with B lineage (*p* < 0.01), myeloid dendritic cells (*p* < 0.01), cytotoxic lymphocytes (*p* < 0.01), CD8 T cells (*p* < 0.01), NK cells (*p* < 0.01), T cells (*p* < 0.01), and neutrophils (*p* < 0.01) (Fig. 9D).

**Fig. 10:**
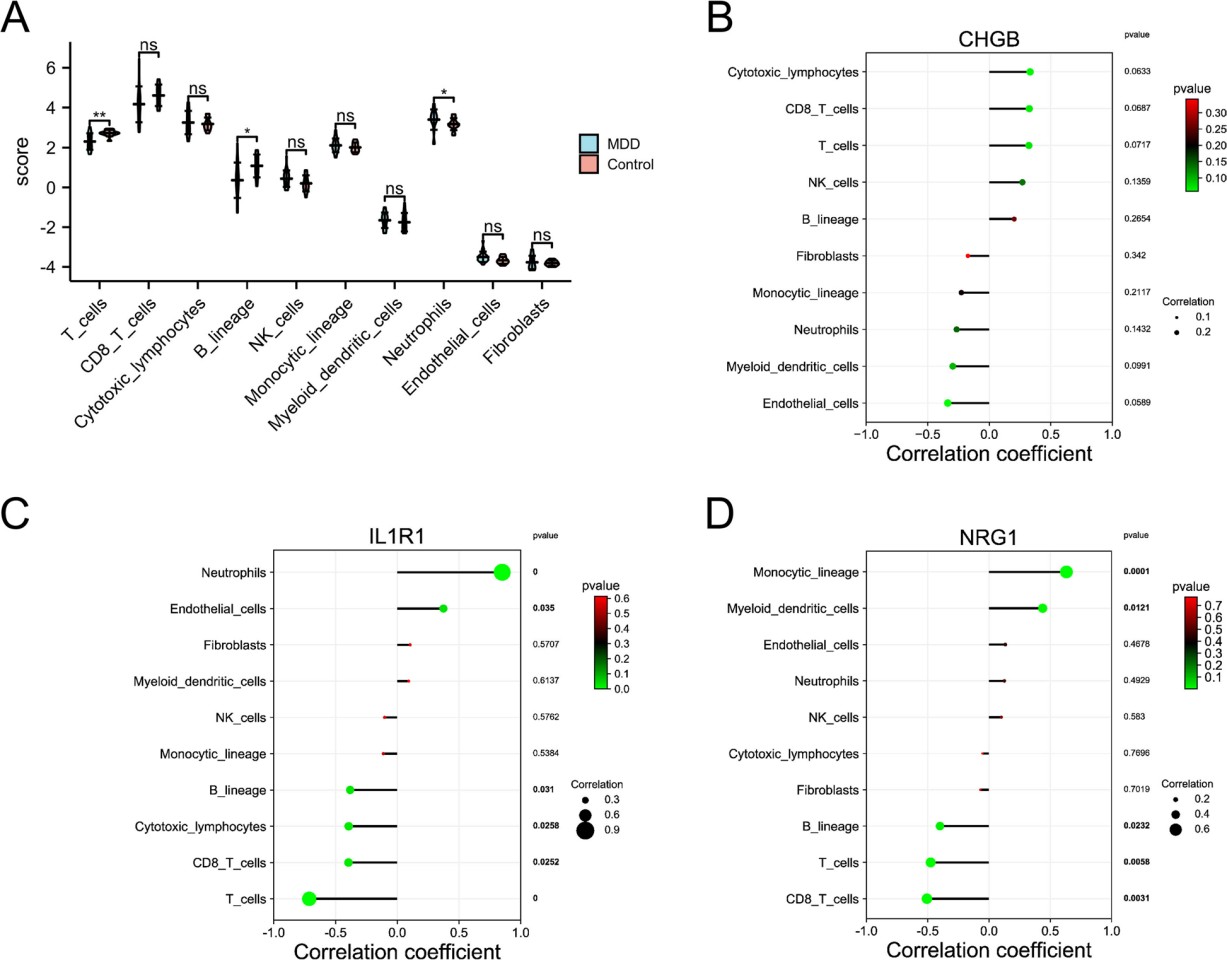
The landscape of immune cell infiltration between control and MDD groups. **A** The violin plots indicate the abundance of immune cell populations in control and MDD groups. Lollipop diagram of a correlation between CHGB (**B**), IL1R1 (**C**), NRG1 (**D**) expression, and immune cell infiltration level

As shown in Fig. 10A, we found the differences in T cells, B lineage, and neutrophils between MDD and control groups, suggesting that immune cell infiltrations play a vital role in the pathogenesis of MDD. CHGB does not correlate with immune cells (Fig. 10B). IL1R1 was positively correlated with neutrophils (*p* < 0.01) and endothelial cells (*p* < 0.05), but negatively correlated with T cells (*p* < 0.01), CD8 T cells (*p* < 0.05), cytotoxic lymphocytes (*p* < 0.05), and B lineage (*p* < 0.05) (Fig. 10C). NRG1 was positively correlated with monocytic lineage (*p* < 0.01) and myeloid dendritic cells (*p* < 0.05), but NRG1 was negatively correlated with CD8 T cells (*p* < 0.05), T cells (*p* < 0.05), and B lineage (*p* < 0.05) (Fig. 10D). These results showed that IL1R1 and NRG1 were correlated with immune cell infiltrates both in AD and MDD.

## Discussion

AD and MDD are both common in older adults and often occur together [35]. Previous reports revealed that prior depression increases the risk of dementia; however, their interconnectedness is complex and not well understood. In addition, both MDD and AD are affected by genetic factors [36, 37], and shared genetic risk factors may explain some of the associations between these diseases [38]. Immune system disorders are considered risk factors in a variety of neurological disorders, including AD, MDD, and Parkinson’s diseases [29]. In recent years, some researchers have identified biomarkers associated with immune infiltration for AD or MDD patients by bioinformatics analysis. For example, four immune-related genes were identified as diagnostic biomarkers of MDD and were associated with immune infiltration [39]. A recent study has identified and verified six immune-related genes in AD patients [40]. Although a recent study identified five hub genes (DYNCIHI, MAPRE3, TTBK2, ITGBI, and WASL) that could act as biomarkers for the diagnosis and treatment of MDD and AD [41]. However, the mechanisms underpinning the role of IRGs in AD and MDD remain unclear. Our study aimed to identify potential shared IRGs and correlated immune cell infiltrations between AD and MDD. In our study, for the first time, we identified 3 shared DEIRGs (IL1R1, CHGB, and NRG1) between MDD and AD by integrated analyses of GEO datasets. Functional enrichment analysis revealed that MDD and AD shared some of the common pathways: primary immunodeficiency, cytokine-cytokine receptor interaction, and JAK-STAT signaling pathway, etc. Furthermore, correlation analysis revealed that both IL1R1 and NRG1 expression are significantly associated with neutrophils, endothelial cells, and myeloid dendritic cell infiltrations in AD and MDD.

Interleukin-1 receptor-like 1 (IL1R1) is an immune-related gene and has been involved in the pathology of multiple sclerosis and experimental autoimmune encephalomyelitis [42]. IL1R1 was higher in the epilepsy group than in the control group [43]. A previous study indicated that mRNA expression of IL1R1 was significantly up-regulated in the lymphocytes of patients with MDD [44]. Furthermore, a growing amount of evidence indicated that inactivation of IL1R1 signaling in the experimental models of central nervous system diseases, including multiple sclerosis, Parkinson’s disease, AD, and amyotrophic lateral sclerosis, resulting in decreased neuroinflammation and delayed disease progression [45]. Consistent with these previous studies, our findings indicated that IL1R1 expression was up-regulated both in MDD and AD patients. Thus, IL1R1 may be a shared genetic risk for MDD and AD.

Chromogranin B (CHGB) is a member of the chromogranin gene family and has been identified as a potential biomarker related to the risk of schizophrenia [46, 47]. It has been reported that CHGB may be important for the regulation of synaptic transmission to promote the occurrence and progression of AD [48]. And CHGB is a potential biomarker for human hippocampal pathways [49]. Based on these studies, we speculated that CHGB may be involved in the pathogenesis of AD and MDD.

Neuregulin-1 (NRG1) is a paracrine growth factor and has been involved in synaptic plasticity and neural development, and plays a vital role in psychiatric diseases, such as bipolar disorder, schizophrenia, and depression [50, 51]. NRG1 promotes hippocampal long-term depression induction in adult mice [52]. Nedd4l-mediated downregulation of NRG1 in the medial prefrontal cortex induced depression-like phenotypes mice in chronic social defeat stress [53]. Moreover, NRG1 plays a vital role in the development and plasticity of the brain. For example, pretreatment with NRG1 protects neuronal cells against damage via inhibiting CoCl2-induced accumulation of p53 stability [54]. NRG1 improves neuropathology and cognitive deficits in AD mice [55]. NRG1 may be implicated in the pathophysiology of AD, and regulation of NRG1 level may represent a novel target in AD [56]. It has been demonstrated that the NRG1 signaling pathway may impact the pathological process of AD, and it may serve as a potential target for the prevention and treatment of AD [57]. Based on these studies, NRG1 is a possible shared gene for MDD and AD.

Immune cell infiltration plays an important role in AD and MDD. For example, increased neutrophil-to-lymphocyte at admission was indicated to be associated with post-stoke depression [58]. Endothelial mitochondrial dysfunction contributes to the progression of neurovascular dysfunction in dementia and AD [59]. Our correlation analysis revealed that the expression of IL1R1 and NRG1 had a negative or positive correlation with some immune cells (neutrophils, endothelial cells, and myeloid dendritic cell infiltrations) both in AD and MDD.

However, our current study has some limitations. First, for the diagnosis of MDD and AD patients, the sample tissue sources are different. Future studies need to use the same sample source (e.g., blood, tissue samples and/or cell lines) to verify the expression of shared genes. Second, larger numbers of clinical samples are needed to validate the expression of shared genes.

## Conclusion

In conclusion, the present study was focused on the identification of DEIRGs in AD and MDD, and three shared genes (IL1R1, CHGB, and NRG1) had a good diagnostic value both in MDD and AD. Furthermore, IL1R1 and NRG1 have correlated with immune infiltrates both in AD and MDD, which may be used as novel targets for immunotherapy both in MDD and AD patients.

## Data Availability

The datasets analyzed during the present study are available in the GEO database: GSE76826 (https://www.ncbi.nlm.nih.gov/geo/geo2r/?acc=GSE76826); GSE5281 (https://www.ncbi.nlm.nih.gov/geo/geo2r/?acc=GSE5281); GSE98793 (https://www.ncbi.nlm.nih.gov/geo/geo2r/?acc=GSE98793); GSE132903 (https://www.ncbi.nlm.nih.gov/geo/geo2r/?acc=GSE132903).
